# Evolution-guided evaluation of the inverted terminal repeats of the synthetic transposon *Sleeping Beauty*

**DOI:** 10.1038/s41598-018-38061-w

**Published:** 2019-02-04

**Authors:** Barbara Scheuermann, Tanja Diem, Zoltán Ivics, Miguel A. Andrade-Navarro

**Affiliations:** 1 Faculty of Biology, Johannes Gutenberg University of Mainz, 55128 Mainz, Germany; 2 Division of Medical Biotechnology, Paul Ehrlich Institute, Langen, Germany

## Abstract

*Sleeping Beauty* (SB) is a synthetic Tc1/*mariner* transposon that is widely used for genetic engineering in vertebrates, including humans. Its sequence was derived from a consensus of sequences found in fish species including the Atlantic salmon (*Salmo salar*). One of the functional components of SB, the transposase enzyme, has been subject to extensive mutagenesis yielding hyperactive protein variants for advanced applications. The second functional component, the transposon inverted terminal repeats (ITRs), has so far not been extensively modified, mainly due to a lack of natural sequence information. Importantly, as genome sequences become available, they can provide a rich source of information for a refined molecular definition of the functional components of these transposons. Here we have mined the *Salmo salar* genome for a comprehensive set of transposon sequences that were used to build a refined consensus sequence. We synthetically produced the new consensus ITR sequences and used them to build a new transposon, the performance of which has been tested in cell-based transposition assays. The consensus sequence did not support enhanced transposition, suggesting alternative mechanisms responsible for the preferential amplification of these sequence variants in the salmon genome.

## Introduction

Transposable elements of the Tc1/*mariner* superfamily have a length of 1300–2400 bp, comprising a coding region for a transposase in the middle of the transposon and one inverted terminal repeat (ITR) on each side^1^. The ability of transposons from this family to change their position within a genome is defined by these two functional components in that the transposase recognizes the transposon sequence at the ITRs, binds that sequence, excises the whole transposon from its place and inserts it to another spot in the genome by a cut-and-paste mechanism^2^.

Transposons are one of the most efficient genomic engineering tools as they directly insert their genetic cargo into genomes. In particular, the *Sleeping Beauty* (SB) transposon system has been extensively used for genetic engineering, including germline transgenesis in animals, insertional mutagenesis screens and gene therapy (reviewed in^3–10^). SB is a synthetic transposon system consisting of a transposase whose sequence matches a consensus derived from multiple Tc1/*mariner* transposon sequences found in 8 fish species including *Salmo salar* (Atlantic salmon^11^). The ITR sequences that are currently used in conjunction with the SB transposase have been derived from a naturally occurring single element isolated from the *Tanichthys albonubes* genome (the white cloud mountain minnow, not a salmonid species) and are 3.8% divergent from the salmonid consensus^12^. In an attempt to derive hyperactive transposase variants for advanced genetic engineering, amino acid substitutions spanning almost the entire SB transposase have been screened for enhanced catalytic activity. The most hyperactive SB transposase version currently available, SB100X, displays a ~100-fold hyperactivity and contains 9 amino acid substitutions when compared to the originally resurrected transposase^3^.

As with the transposase, although to a lesser extent, mutagenesis of the SB ITR sequences has been undertaken with the aim to increase the efficiency of transposition. For example, replacement of 4 base pairs in the right ITR and flanking both ITRs by a TATA sequence motif (as opposed to the canonical TA flanks) in the pT2 transposon vector resulted in ~3-fold increase in transposition over the first-generation ITR sequence^13^.

The sequencing of the complete genome of *Salmo salar*^14^ and, particularly, the recent very high quality assembly^15^), gives us now the chance to update the consensus of this particular Tc1/*mariner* subfamily (called Tss1) using all sequence instances found in this genome, under the assumption that this consensus might have been optimized for efficiency in a process of selection and evolution.

## Results

### Refining the consensus of the Tss1 transposon

**Figure 1:**
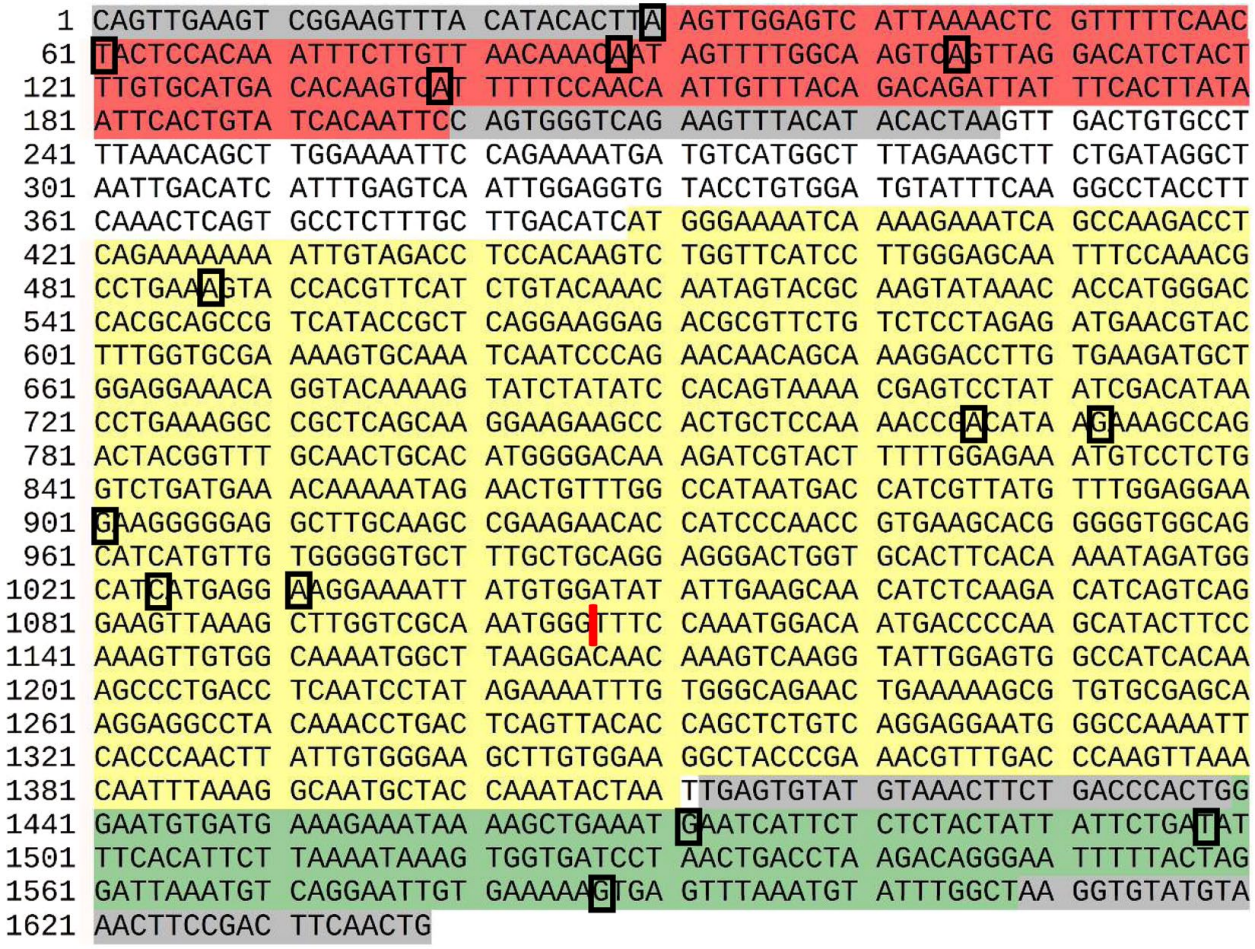
DNA sequence of the *Sleeping Beauty* transposon. The total length of the sequence is 1638 nt. The transposase coding region (yellow) is flanked by one left inverted repeat (red) and one right inverted repeat (green) with the respectively transposase binding regions (gray)^11^. The black boxes and the red line represent positions that were different in the consensus, and one insertion, respectively (see text and Methods for details; Figs S1–S3).

Variants of the Tss1 family transposons^12^ in the full genome of *Salmo salar* were obtained by sequence similarity search using the full sequence of SB or fragments (Fig. 1) as query (see Methods for details). Searching with the full SB sequence, we identified 67 hits. To study the robustness of the consensus we created a restricted set with the sequences having at least 90% identity to SB (39 sequences). A two-step search was also tried using first the transposase coding sequence and then verifying that there were hits to the inverted repeats in its genomic flanking regions (112 hits; transposase trim search; see Methods for details). Also, for each of the three features investigated (left repeat, right repeat, transposase coding region) a separate search and analysis of the hits was attempted.

We created consensus sequences by aligning each set of hits and taking the nucleotides conserved in at least at 60% of the sequences (see Methods for details). Even using this somehow restrictive level, the similarity between the results obtained from the four strategies to collect hits described above was near 100% (Figs S1–S3).

Only a few differences were observed between the consensus and the sequence of SB used as query. For the left repeat there were four differences (Fig. S1) identical in the four consensus sequences at positions 31, 61, 88 and 105, and position 138 was found to be variable except for the two-step search consensus. The search with the repeat only (which resulted in 781 hits) has a 55.9% of C, very close to our threshold, with 43.7% of A. All these differences were found outside the transposase binding regions and thus would not be expected to change the recognition of the sequence by the enzyme.

For the right repeat we observed three differences (Fig. S2) identical in the four consensus sequences at positions 60, 87 and 176. As above, all these differences were found outside the transposase binding regions.

We identified a few changes for the coding region of the SB transposase (Fig. S3). Particularly interesting for this segment of the transposon sequence is whether changes in the sequence affect the resulting amino acid sequence of the protein. All nucleotide changes were observed in the third positions of codons in the four consensus sequences at positions 99 (A to G; codon AAA to AAG: synonymous change for Lys), 378 (A to C; CGA to CGC; synonymous for Arg), 384 (G to A; AAG to AAA; synonymous for Lys) and 513 (G to A; AAG to AAA; synonymous for Lys).

At position 636, only the two consensus from the searches using the complete SB sequence show a change (C to A; ATC to ATA; synonymous for Ile).

At position 643 we did not find any nucleotide at more than 60% frequency; for example, using the 244 hits from the transposase sequence alone the frequency was A 43% > G 22% > C 19% > T 14% (with similar frequencies for the other three consensus). This is the first nucleotide of an AAG codon, thus, the most frequent amino acid in the consensus matches the original one in the SB.

At position 719 we observed a conserved insertion (A) in all consensus; this is in the coding region of the transposase, which changes the frame, in agreement with the sequence of a hyperactive variant (SB100X^16^), for which the structure of the catalytic domain of the transposase was solved (amino acids 114–340; PDB:5CR4^17^).

In summary, the differences we observed in the coding part of SB do not change the amino acid sequence of the transposase, and therefore might be reflecting neutral variation of the ancestral transposase.

**Figure 2:**
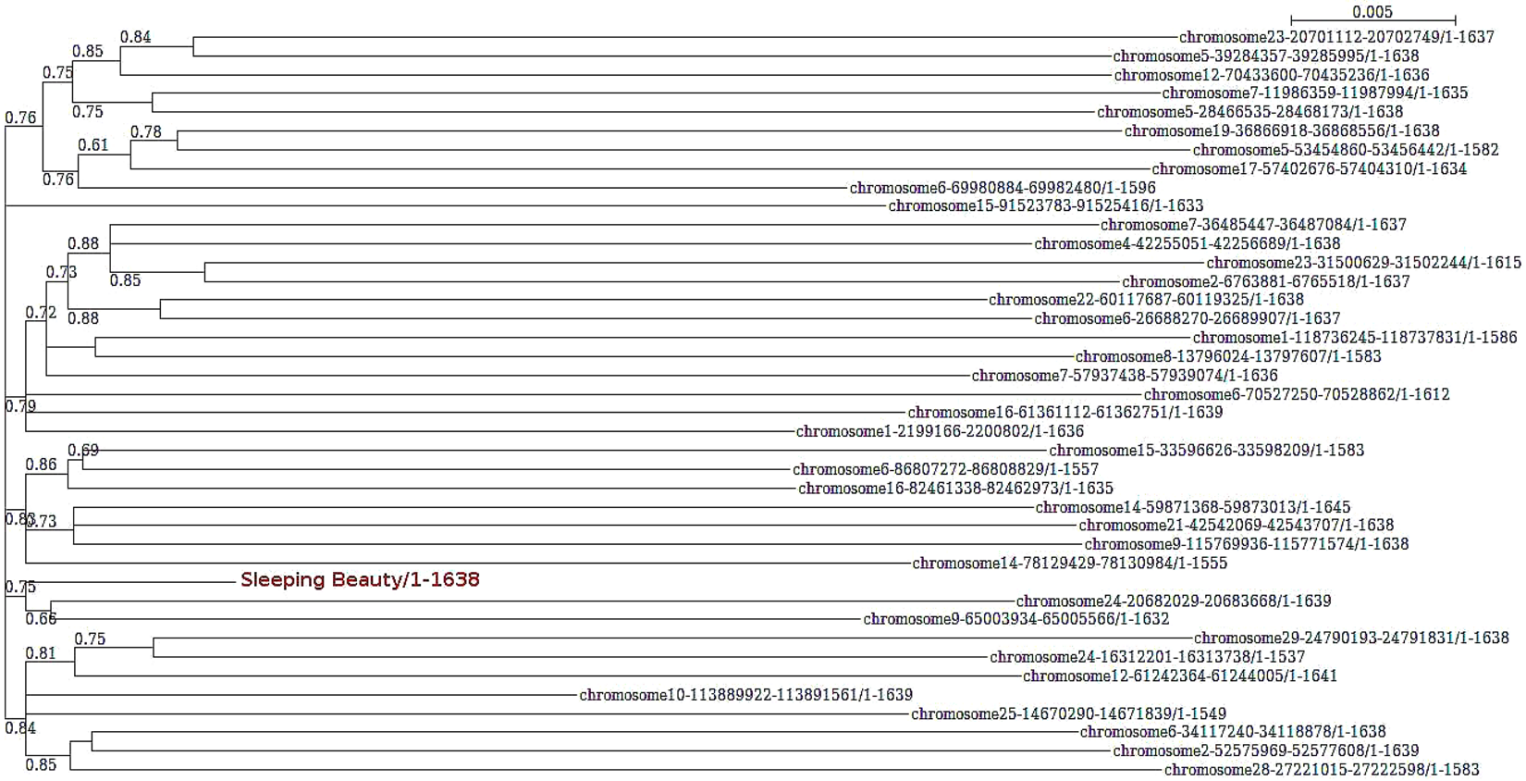
Phylogenetic tree of Tss1 hits. The tree was constructed from the multiple sequence alignment of SB and the 39 nucleotide sequences found when searching with SB (more than 90% identical to SB), using Seaview with the HKY85 substitution model^28^. Branch support values (aLRT-SH-like) over 0.5 are represented.

The phylogenetic trees for each set of sequences did not indicate clear separation of subfamilies, suggesting again that the variation we found was due to neutral divergence from the ancestral functional sequence (Fig. 2). The short branch of SB with respect to the other sequences suggests that its sequence was already an approximation to the ancestral transposon, and explains the few differences we found between our consensus and its sequence.

### Experimental test of an SB ITR variant

We hypothesized that the consensus found for ITRs in the *Salmo salar* genome could correspond to sequences with higher efficiency of transposition. To test this hypothesis, we incorporated the newly generated sequence information into the ITRs of a genetically tagged SB transposon, and evaluated the performance of this new transposon against the state-of-the-art pT2 transposon vector. We synthetically produced the ITRs carrying G in position 31, C in position 61, T in position 88 and G in position 105 in the left ITR (Fig. S1), and A in position 60, C in position 87 and C in position 176 in the right ITR (Fig. S2). The synthetic ITR sequences were used to flank a puromycin resistance gene in a way that the resulting transposon is indistinguishable from a pT2-based transposon except the 7-bp replacements within the ITRs.

**Figure 3:**
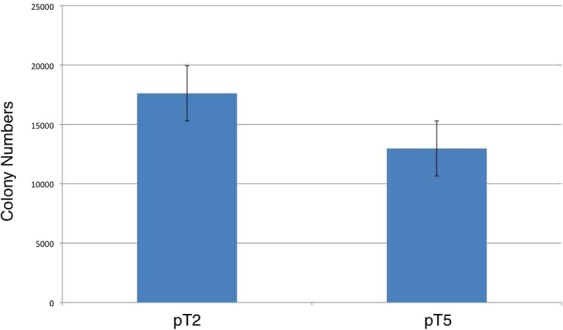
Relative transposition efficiencies. The newly designed consensus ITR sequences have been incorporated in the pT5 transposon vector, whose potency to mediate transposition was evaluated against the state-of-the-art pT2 SB transposon vector. Transposon vectors carrying a puromycin resistance gene were cotransfected with a source of SB100X transposase into human HeLa cells, and puromycin-resistant cell colonies were counted. n = 4, error bars represent standard error of the mean.

The newly generated transposon (hereby designated pT5) and the pT2-based transposon were transfected into cultured human HeLa cells together with the SB100X transposase or catalytically inactive SB transposase as negative control, and puromycin-resistant colonies containing transposon integrations in the HeLa cell genome (Fig. S4) were counted. Colony numbers obtained with pT5 were slightly lower than those obtained with pT2 (Fig. 3), indicating that the 7-bp difference in the ITR sequence is unlikely to have contributed to the relative success of this sequence variant in the *Salmo salar* genome.

## Discussion

We hypothesized that the consensus of ITRs in the *Salmo salar* genome might correspond to a sequence variant associated with increased transpositional potency as compared to SB. We tested this hypothesis by building a new transposon carrying the consensus ITR sequences and testing its performance in transfected human cells. However, the consensus ITRs did not support higher transposition rates, suggesting that mechanisms that are independent from these 7 bps were responsible for the accumulation of these elements.

One potential mechanism that could have played a role in amplification of these sequence variants can be the chromatin environment and/or other structural features of the loci from which transposition took place (that is, a mechanism that has an impact on the efficiency of transposition independent from the primary DNA sequence of the transposon ITRs). Indeed, a model has been proposed, in which CpG methylation and subsequent chromatin condensation aids transposase binding at the synaptic complex formation step^18^. Because heterochromatin formation results in tight packaging of DNA and histones, DNA sites that are usually far away from each other; for example, the transposase binding sites in the ITRs, might be brought closer together. Interestingly, it was found that the enhancing effect of CpG methylation is not restricted to SB but is common to those Tc1/*mariner* transposons, including SB, *Frog Prince* and *Minos*, that have a characteristic ITR structure^19^. Thus, it may be that certain sequence variants of *Salmo salar* transposons were preferentially amplified, because they were situated in a chromatin context supporting higher levels of transposition.

Another possible explanation would be that the fitness landscape of SB sequences has a flat maximum around the consensus that allows for large variation around it without changes of efficiency approaching that of a quasi-species distribution^20^. This model predicts that if the SB sequence is already close enough to that flat maximum, it would not be possible to improve its sequence further.

In any case, we have demonstrated that it is possible to apply an evolutionary strategy to explore transposon function. This might be useful for the study of the function of other molecular elements, as they become technically available, and as new genomic information is obtained.

## Methods

### Genomic analyses

We analysed the recent high quality assembly of the complete genome of *Salmo salar*^15^ to research the occurrence and variation of previously characterized transposons. The sequences of its chromosomes were downloaded from the NCBI GenBank: ftp://ftp.ncbi.nlm.nih.gov/genomes/Salmo_salar/Assembled_chromosomes/seq/ (visited on Feb. 12, 2016; version ISCAG v2).

To identify instances of Tss1 transposons we searched the genome with BLAT^21^ using full or parts of SB as query (Fig. 1) with very permissive parameters (-stepSize = 5, -repMatch = 10000, -minScore = 0, -minIdentity = 0, -fastMap, -oneOff = 1). We filtered the hits by length discarding hits deviating more than 10% of the length of the query. One search used the full-length sequence (1638 nt). Considering that not all transposons are “complete” and maintain all the functional parts intact, a search was performed with each of the three parts of the transposon separately, that is, each of the two inverted repeats including the transposase binding regions (227 nt) and the transposase coding region (1022 nt).

In addition, a two-step search was tried (transposase trim search) where hits for the transposase region were verified for the presence of the repeats to avoid matching homologous proteins. Basically, each hit was extended 500 nt on each side, and the hit was taken for further analysis only if the repeats were detected on each extended region. Sequence duplicates were removed.

We followed the criteria proposed by^22^ for the selection of an appropriate program for multiple sequence alignment. Multiple sequence alignment based on fast Fourier transform (MAFFT^23^) appeared to be the best suited for our purposes. MAFFT is especially suited for larger datasets. Default settings (FFT-NS-2, the fast progressive method) or the L-INS-i method were used when the sets were either smaller or larger than 200 sequences, respectively.

Multiple sequence alignments were visualized, analysed and edited using Jalview^24^. Manual examination of alignments was required to eliminate sequence hits with a high number of insertions or deletions; such sequences were not considered in order to acquire consistent results. Subsequently, the sets of annually curated results were further processed in Seaview^25^.

The consensus from each sequence set was calculated based on the most frequent nucleotide at each position of the alignment with Seaview (threshold value 60% frequency). The choice of a strict definition of sequence conservation was intended to focus the identification of conserved positions on those most relevant. Variable positions (identified with an N) were studied separately. Phylogenetic trees were made using PhyML (as integrated in SeaView)^26^.

Protein coding regions were translated with the translate tool from ExPASy^27^.

### Cloning and functional testing of a new ITR variant

The left and right ITR consensus sequences shown in Figs 2 and 3 have been synthetically manufactured by Genewiz (South Plainfield, NJ, USA), and cloned at the *Sac*I/*Hin*dIII and *Bgl*II/*Sal*I sites of pT2/HB^13^. An 1481-bp *Hinc*II fragment carrying an SV40 promoter-driven puromycin resistance gene was then cloned into the *Eco*RV site between the ITRs. The resulting plasmid (pT5/HB-puro) was transfected into 3 × 10^5^ human HeLa cells together with the CMV(CAT)T7-SB100X expression plasmid^16^ or the pCMV-D3 catalytically inactive SB transposase variant^16^ as negative control. The transfected cells were put under puromycin selection, and antibiotic-resistant cell colonies were counted 2 weeks post-transfection and compared to colony numbers obtained with pT2/HB-puro reference control.

## Supplementary information

Supplementary Material

Supplementary File S1

## Data Availability

All sequence data analysed during this study are public. The datasets generated during this study are available from the corresponding author on request.
