# Supplementary material for: Evolution-guided evaluation of the inverted terminal repeats of the synthetic transposon Sleeping Beauty
Source: Sci Rep. 2019 Feb 4;9:1171. doi: 10.1038/s41598-018-38061-w (PMC6362248; doi:10.1038/s41598-018-38061-w)
Supplement: Supplementary file 1 — Supplementary Material [file 41598_2018_38061_MOESM1_ESM.pdf]

Supplementary material for:

Evolution-guided evaluation of the inverted terminal repeats of the synthetic transposon *Sleeping Beauty*

Barbara Scheuermann[1], Tanja Diem[2], Zoltán Ivics[2,*] and Miguel A. Andrade-Navarro[1,*]

[1]Faculty of Biology, Johannes Gutenberg University of Mainz, 55128 Mainz, Germany

[2]Division of Medical Biotechnology, Paul Ehrlich Institute, Langen, Germany

[*]Equal contribution

SUPPLEMENTARY FIGURES

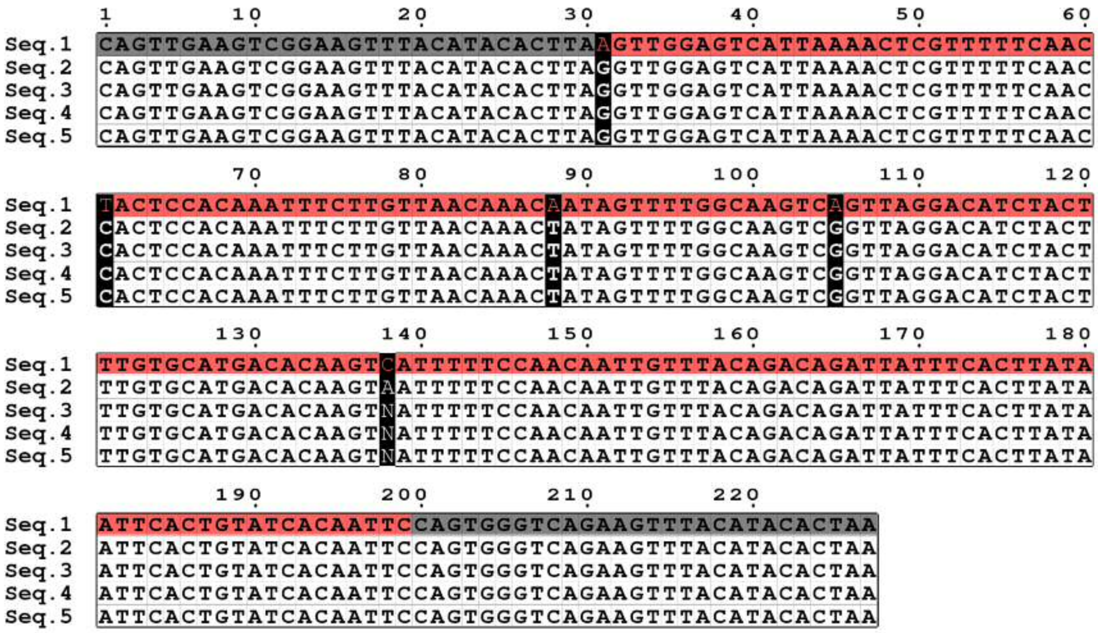

Figure S1. Consensus of the Tss1 left inverted repeat. The DNA sequence of the left inverted repeat of SB (Seq.1) is aligned to consensus sequences built from hits obtained by searching the genome of *S. salar* for similar sequences with BLAT using as query full-length SB or fragments. Consensus were derived from the transposase trim two-step search (112 hits, Seq.2), from the search using full-length SB either with permissive restraints (67 hits, Seq.3) or with high restrictions (39 hits, Seq.4), and from the search using only the sequence displayed in this figure (781 hits, Seq.5). Black background indicates differences between SB and the different consensus. See Methods for details.

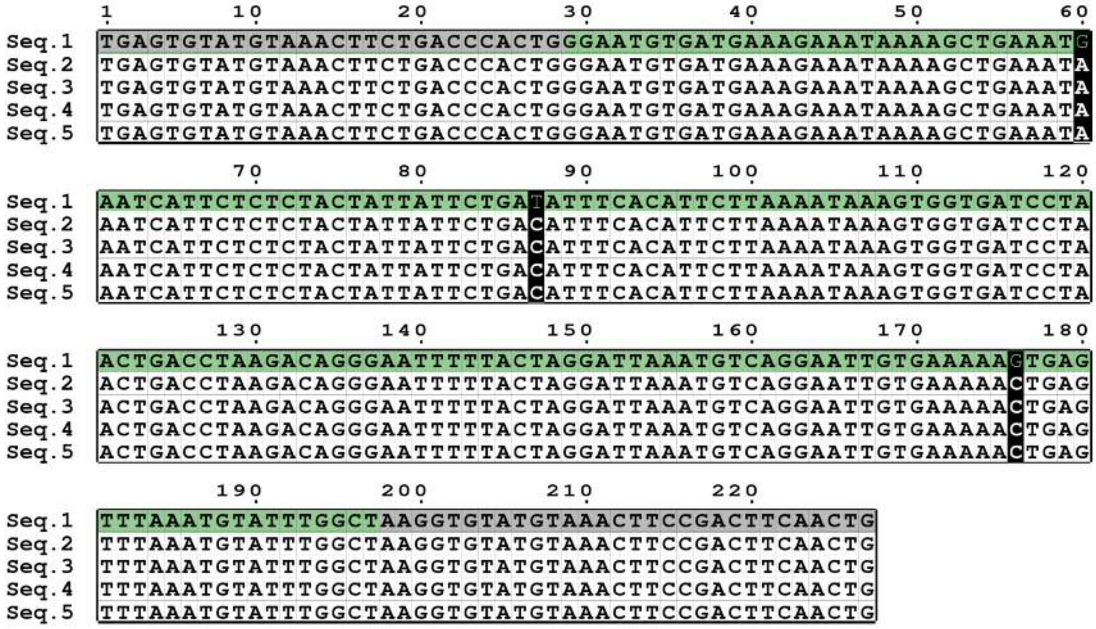

Figure S2. Consensus of Tss1 right inverted repeat. Original sequence (Seq.1) aligned to consensus sequences obtained as defined as in **Fig. S1**. A search using the SB fragment displayed in this figure (Seq.1) resulted in 860 hits, which were used to build the Seq.5 consensus.

Figure S3

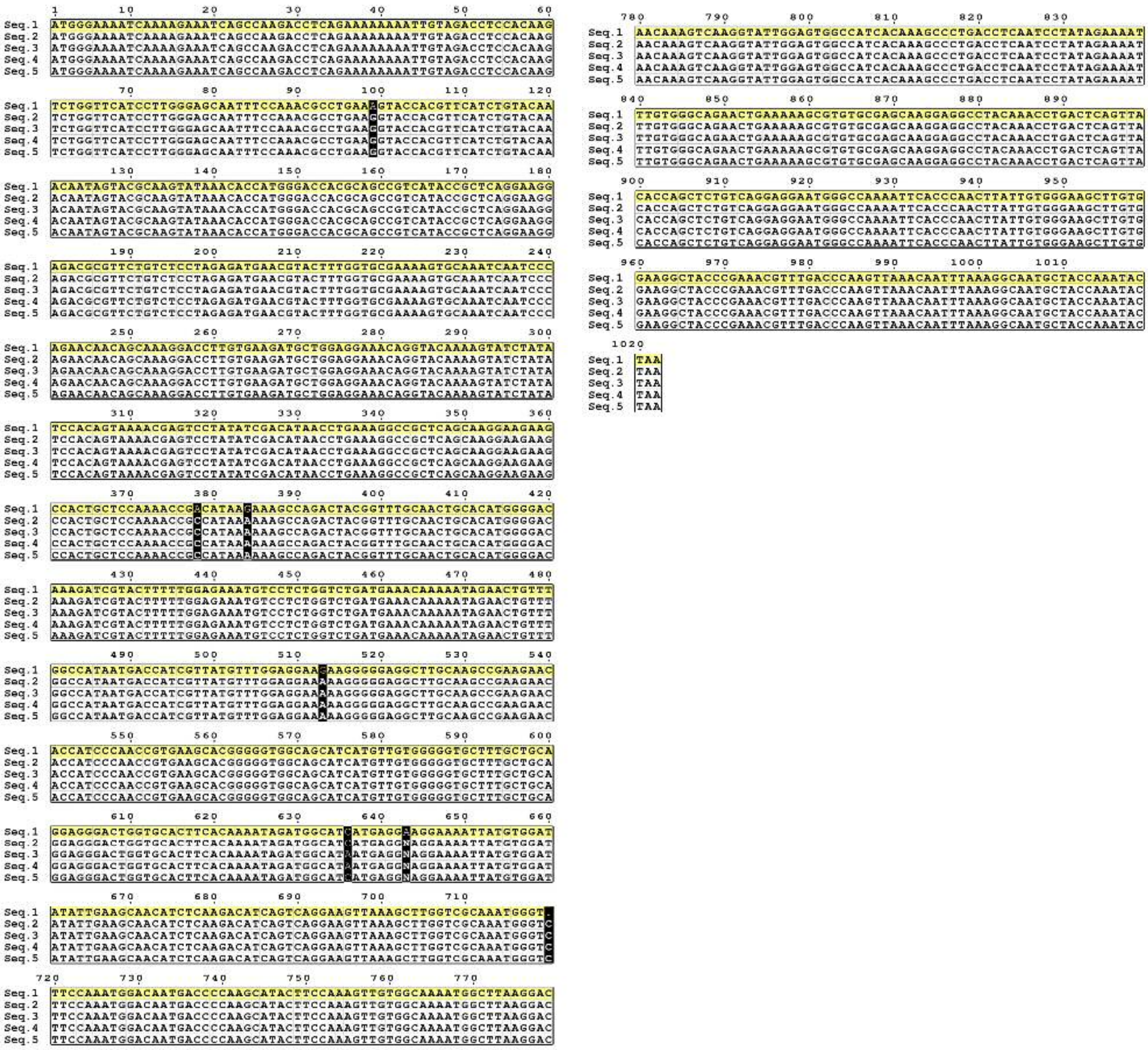

Figure S3. Consensus of Tss1 transposase coding region. Original sequence (Seq.1) aligned to consensus sequences obtained as defined as in **Fig. S1**. A search using the SB fragment displayed in this figure (Seq.1) resulted in 244 hits, which were used to build the Seq.5 consensus.

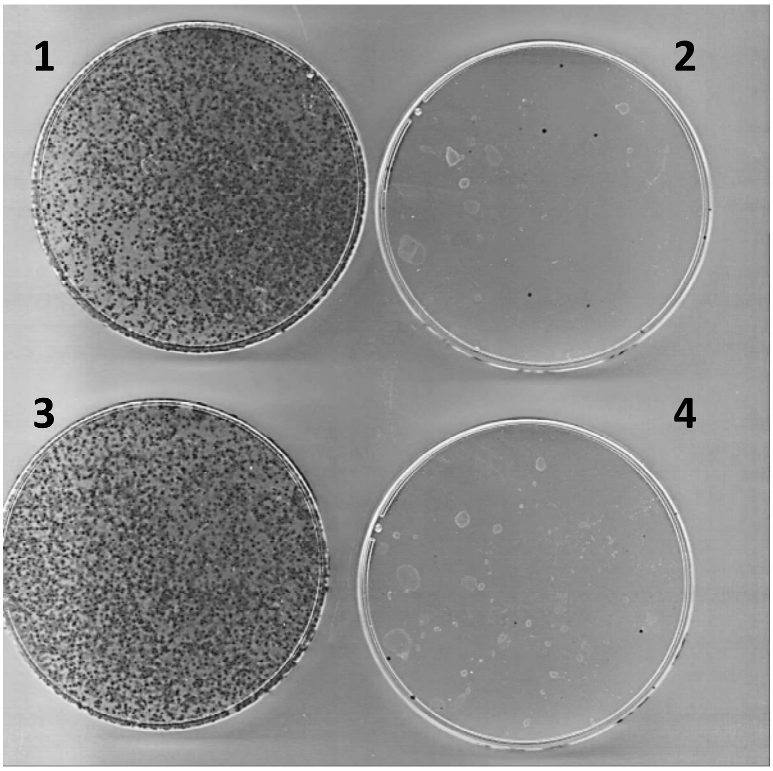

1 – pT2/HB-puro + CMV(CAT)T7-SB100X

2 – pT2/HB-puro + CMV-D3

3 – pT5/HB-puro + CMV(CAT)T7-SB100X

4 – pT5/HB-puro + CMV-D3

Figure S4. Transposition assay in human HeLa cells. The pT5/HB-puro and pT2/HB-puro transposon donor plasmids (200 ng plasmid DNA) were transfected together with either CMV(CAT)T7-SB100X or catalytically inactive CMV-D3 (50 ng plasmid DNA) into $3x10^5$ human HeLa cells. Twenty percent of the transfected cells were put under puromycin selection 48 hours post-transfection, and antibiotic-resistant cell colonies were counted and photographed 2 weeks post-transfection. One representative experiment is shown.

SUPPLEMENTARY FILES

Supplementary File S1. A zip file containing the alignments used for producing the consensus used in this manuscript.
